# Impact of Thrombocytopenia in Patients With Atrial Fibrillation Undergoing Left Atrial Appendage Occlusion: A Propensity-Matched Comparison of 190 Consecutive Watchman Implantations

**DOI:** 10.3389/fcvm.2021.603501

**Published:** 2021-04-09

**Authors:** Xiaochun Zhang, Qinchun Jin, Jialu Hu, Dehong Kong, Cuizhen Pan, Dandan Chen, Shasha Chen, MIngfei Li, Daxin Zhou, Junbo Ge

**Affiliations:** ^1^Department of Cardiology, Zhongshan Hospital, Fudan University, Shanghai, China; ^2^Research Unit of Cardiovascular Techniques and Devices, Chinese Academy of Medical Sciences, Beijing, China; ^3^National Clinical Research Center for Interventional Medicine, Shanghai, China; ^4^Department of Cardiac Echocardiology, Zhongshan Hospital, Fudan University, Shanghai, China

**Keywords:** atrial fi, left atrial appendage occlusion, thrombocytopenia, bleeding, stroke

## Abstract

**Objectives:** The purpose of this study was to provide data on the long-term efficacy and safety of left atrial appendage occlusion (LAAO) in patients with atrial fibrillation (AF) and chronic thrombocytopenia (cTCP).

**Methods:** Between January 2016 and December 2018, a total of 32 AF patients with thrombocytopenia (platelet count <100^*^10^∧^9/L) undergoing LAAO at our center were identified and their outcomes were compared with a propensity-matched cohort (match ratio 1:5) of patients without cTCP who had also been indicated for LAAO.

**Results:** Between the cTCP patients and the control group, no difference was found on the incidence of stroke (0 vs. 3.13%, *p* = 0.592), systematic thromboembolisation (0 vs. 0.63%, *p* > 0.9) and device-related thrombus (DRT) (3.13 vs. 2.50%, *p* > 0.9). Major (12.50 vs. 3.75%, *p* = 0.065) and minor bleeding (15.63 vs. 1.25%, *p* = 0.002) was more frequent in cTCP patients but no statistical difference was reached in major bleeding. Moreover, thrombocytopenia was also identified as an independent predictor of any bleeding events (OR: 8.150, 95% CI: 2.579–25.757, *p* < 0.001), while an inverse relationship between higher absolute platelet count and stroke events was revealed (OR: 1.015; 95% CI: 1.002~1.029, *p* = 0.022). However, in both groups we saw a significant reduction in observed annualized rates of non-procedural complications compared with the predicted values. In the cTCP and control groups, clinical thromboembolism was reduced by 100 and 74.32%, and major bleeding by 42.47 and 71.67%, respectively.

**Conclusion:** Our preliminary results indicate that LAAO using the Watchman device could be a safe and effective means of preventing stroke in AF patients with or without thrombocytopenia, but bleeding complications should be monitored intensively in cTCP patients.

## Introduction

Thrombocytopenia, usually defined as a platelet count lower than 100^*^10^∧^9/L, is estimated to account for ~6–24% of patients with AF (1, 2). Thrombocytopenia does not protect against thromboembolic events in adult patients, especially in the presence of an AF comorbidity (3, 4). Therefore, given the increased mortality rates, thrombocytopenic patients were excluded from most randomized controlled trials using non-vitamin-K antagonist oral anticoagulants (NOACs) or warfarin (5–7). In addition, several observational studies have demonstrated a higher incidence of bleeding events among thrombocytopenic patients (8–10). To date, the current guidelines for AF management have addressed no definite recommendations for this special population (11).

Given their chronic exposure to stroke, high bleeding risk, and therapeutic decisions to consider, there is a significant need for management in AF patients with thrombocytopenia in order to counterbalance the benefits with the risks of complications. Recently, LAAO has been acknowledged as a reliable alternative for cardioembolic stroke prevention with an efficacy equal to anticoagulation therapy (12). A significant continued reduction in bleeding rates has also been documented in the device group, particularly after the procedural period (12). However, there is limited data on LAAO in patients with prior thrombocytopenia. The purpose of the present study was to prospectively estimate the safety and efficacy of LAA implantation during mid- to long-term follow-up.

## Materials and Methods

In this observational study, we prospectively evaluated an unselected cohort of AF patients indicated for LAAO between January 2016 and December 2018. Patients were excluded if they were receiving LAAO as an adjunctive strategy to another cardiac intervention. The enrolled patients were divided into two groups based on their baseline quantitative platelet activity: the thrombocytopenic group (platelet count <10^*^10^∧^9/L) and the control group (platelet count ≥ 10^*^10^∧^9/L). Informed consent was obtained from all participating patients and the study was approved by the Institutional Review Board of Zhongshan Hospital, Fudan University, Shanghai, China.

Baseline demographic and clinical characteristics including age, gender, types of AF, medical history, CHA2DS2-VASC score, HAS-BLED score and laboratory parameters were collected.

### Implantation Procedure and Antithrombotic Therapy After LAA Closure

Prior to LAA closure, each patient was examined using a transesophageal echocardiogram (TEE) 1 day before the procedure to make an initial assessment on cardiac structure and exclude LAA thrombus. All the patients in our study were implanted with the Watchman device using the right femoral vein approach, following the descriptions detailed in previous literature. After a successful transseptal puncture, heparin (70–100 IU/kg) was administered, followed by an optional supplementary dose to maintain an activated clotting time (ACT) of >250 s. We intensively evaluated each individual's PASS criteria, ensuring that these were met before device release. After the procedure, the patients remained in the hospital for at least 2 days, undergoing TEE examinations to exclude significant pericardial effusion/tamponade or device-related complications.

Typically, patients were discharged upon a planned 3-month course of NOAC therapy using rivaroxaban. Dual antiplatelet therapy (DAPT) was administered for at least another 3 months and aspirin (ASA) monotherapy should be continued in the long term. However, the choice and the duration of antithrombotic therapy could be individualized depending on individual patient history and any previous indications for LAAO.

### Clinical Outcomes

Before discharge, all patients were encouraged to report any adverse events immediately. All patients were required to attend a routine clinic visit to undergo TEE at 3 months, 6 months, and every 6 months afterwards in order to allow for adjustments to medication. This was done in accordance with the standard practice of our institution.

In our study, we recorded adverse clinical events including all-cause death, thromboembolism (stroke, transient ischemic attack [TIA], systemic embolization), bleeding events (major bleeding BARC ≥ type-3a), and device complications according to the Munich consensus (13). Among them, Adverse clinical events were categorized as procedure-related if they occurred within 7 days after implantation or during hospitalization.

The primary outcome of our study included all-cause death, thromboembolism, and major bleeding events. Additionally, rates of minor bleeding and DRT were also obtained and compared. A net adverse endpoint was defined as a composite of major adverse clinical events (death, thromboembolism, major bleeding) and imaging abnormality (DRT or dislocation).

On the other hand, to evaluate the efficacy of LAAO for preventing thromboembolic and major bleeding events, a further comparison between the expected annual rates, deriving from CHA2DS2-VASC and HAS-BLED score system, and the actual annualized event incidence was performed in both cohorts.

### Statistical Analysis

Continuous traits were reported as mean ± standard deviation and compared using Student's *t-*test. Categorical variables was reported as the absolute frequency (percentages) and compared using Pearson's chi-squared test or Fisher's exact test. Adverse endpoints occurring over time among our study population were described through Kaplan-Meier survival curves. A logistic regression analysis was used to obtain the odds ratio (OR) and 95% confidence interval (CI) for the development of different endpoints. Variables with a *p*-value lower than 0.1 in a univariate analysis were then involved in the multivariate model. All data analyses were performed using the SPSS software, version 26.0. A significant statistical difference was considered as a two-sided *p* < 0.05.

In consideration of the non-randomized design of our study, propensity-score (PS) matching was performed via a logistic regression model to derive a group of controls who were free of thrombocytopenia and possessed similar characteristics to the thrombocytopenic cohort. Baseline clinical covariates chosen for matching included age, gender, CHA2DS2-VASC and HAS-BLED score, hypertension, diabetes mellitus, congestive heart failure, previous stroke history, previous bleeding history, and abnormal liver and renal function. We attempted a rigorous nearest-neighbor matching algorithm without replacement, using a caliper width ≤ 0.2. Standardized biases were calculated before and after PSM, among which a value of <0.2 was considered as the indicator of adequate bias reduction.

## Results

**Figure 1:**
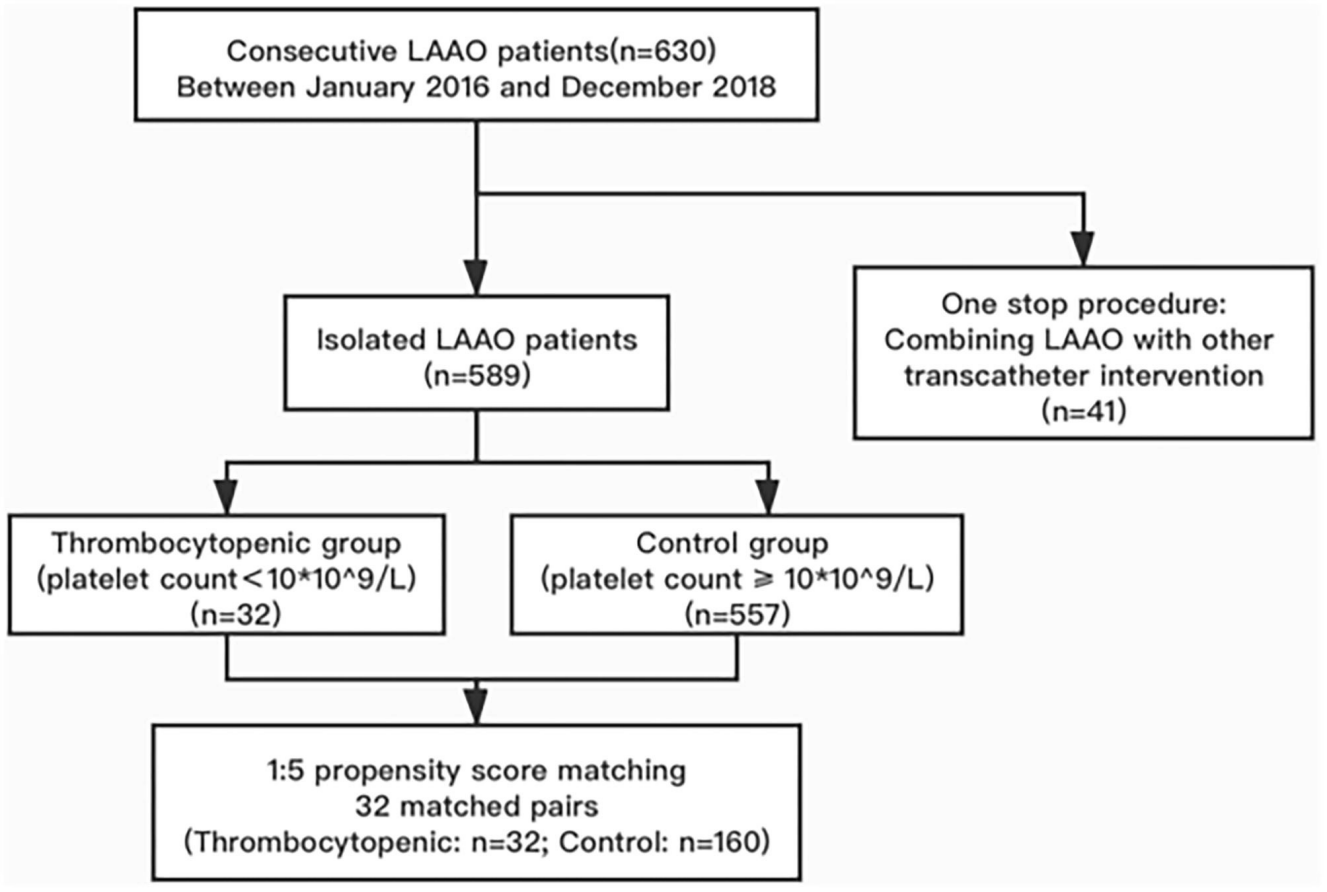
Study flow diagram: the incidence of efficacy and safety outcomes were identified from 32 matched pairs of 193 consecutive patients indicated for LAA implantation.

A total of 630 patients were indicated for LAAO at our clinic between January 2016 and December 2018, among which 41 were categorized as “one-stop” interventions (Figure 1). The remaining 589 patients were divided into the thrombocytopenic group (*n* = 32) and the control group (*n* = 557) based on their antiplatelet count, while 32 matched pairs were successfully selected from PS matching. A standardized bias of <0.2 was observed in all covariates after matching. Patients' baseline characteristics were summarized in Table 1.

**Table 1:** Baseline patient characteristics before and after matching.

**Variables**	**Unmatched cohort**		**Matched cohort**		**Standardized bias before match**	**Standardized bias after match**
	**Thrombocytopenia (*N* = 32)**	**Control (*N* = 557)**	**Thrombocytopenia (*N* = 32)**	**Control (*N* = 160)**		
Age, years	69.44 ± 10.10	68.19 ± 8.90	69.44 ± 10.10	69.20 ± 7.65	0.14	0.03
Gender	8 (25.00)	214 (38.42)	8 (25.00)	43 (26.88)	0.28	0.04
CHA2DS2-VASC	2.81 ± 1.09	3.07 ± 1.52	2.81 ± 1.09	2.84 ± 1.43	0.17	0.02
HAS-BLED	2.72 ± 0.89	2.59 ± 1.18	2.72 ± 0.89	2.79 ± 1.05	0.11	0.07
**Existing comorbidities, n (%)**						
HBP	22 (68.75)	370 (66.43)	22 (68.75)	110 (68.75)	0.05	0
DM	11 (34.38)	111 (19.93)	11 (34.38)	51 (31.88)	0.36	0.05
Previous bleeding history	5 (15.63)	58 (10.41)	5 (15.63)	33 (20.63)	0.17	0.05
Previous thromboembolism	8 (25.00)	246 (44.17)	8 (25.00)	44 (27.50)	0.39	0.06
Congestive heart failure	1 (3.13)	6 (1.08)	2 (3.13)	5 (3.13)	0.03	0
Abnormal liver function	0 (0)	5 (0.01)	0 (0)	0 (0)	0.10	0
Abnormal renal function	1 (3.13)	5 (0.01)	1 (3.13)	2 (1.25)	0.24	0.17
Creatinine, μmol/L	94.47 ± 66.66	85.83 ± 33.54	94.47 ± 66.66	89.24 ± 31.57	0.26	0.17
LVEF	62.77 ± 7.01	62.81 ± 6.83	62.81 ± 6.90	62.83 ± 7.34	0	0

*HBP, high blood pressure; DM, diabetes mellitus; LVEF, left ventricular ejection fraction*.

### Procedural Characteristics

The procedural characteristics of our study population were presented in Table 2. There was no significant difference between the thrombocytopenic and control groups regarding LAA orifice diameter, spontaneous echo contrast (SEC), or LAA flow velocity. Acute procedure success was achieved in all cases, within similar procedure times. Serious peri-device leaks (PDL) were not reported in our study while the incidence of residual leaks was statistically similar between the two groups. Before discharge, an equal proportion of patients in each cohort had been administered with DAPT or NOAC for post-implantation antithrombotic therapy.

**Table 2:** Procedural characteristics.

	**Thrombocytopenic group (*n* = 32)**	**Control group (*n* = 160)**	***P*-value**
LAA ostium diameter (mm)	22.23 ± 3.48	21.49 ± 4.96	0.331
Spontaneous echo contrast in the left atrium	16 (50.00)	70 (43.75)	0.516
LAA flow velocity (m/s)	0.32 ± 0.15	0.36 ± 0.18	0.270
Procedure time (min)	61.91 ± 9.99	62.00 ± 8.50	0.953
Acute procedural success	31 (96.88)	160 (100.00)	0.167
PDL	14 (43.75)	50 (31.25)	0.217
Post-procedure medication			0.699
DAPT	6 (18.75)	36 (22.50)	
NOAC	25 (78.13)	124 (77.50)	

*LAA, left atrial appendage; PDL, peri-device leak; DAPT, dual platelet therapy; NOAC, new oral anticoagulation*.

### Complications and Clinical Outcomes Evaluation

The median follow-up time for the thrombocytopenic group was 2.00 (1.5–3.0) years with a total of 68 patient years, while patients in the control group had a median follow-up duration of 2.00 (1.5–2.6) years, a total of 334.9 patient years. All patients attended routine follow-up visits with TEE examinations within the first 1.5 years. During the 1.5-year follow-up, no statistical difference was observed in the primary endpoints between thrombocytopenic group and control group (12.50 vs. 7.50%, *p* = 0.313) (Table 3). Notably, ischemic stroke (*n* = 5, including one during peri-procedure) and systemic embolization (*n* = 1, peri-procedure) occurred only in patients with normal platelet counts, but none resulted in disability. The rates of major (12.50 vs. 3.75%, *p* = 0.065) and minor hemorrhaging (15.63 vs. 1.25%, *p* = 0.002) were significantly higher in patients with thrombocytopenia but this trend did not reach statistical significance with regard to major bleeding events. Among the bleeding events, both groups reported one case of peri-procedural major bleeding [gastrointestinal bleeding in the thrombocytopenia group and serious pericardial effusion in the control group]. Throughout the follow-up duration of our study, non-procedural major bleeding occurred in 3/32 patients in the thrombocytopenic group (upper gastrointestinal bleeding [*n* = 2], hematuria [*n* = 1]) and 5/160 patients in the control group (upper gastrointestinal bleeding [*n* = 2], hematuria [*n* = 2], lower gastrointestinal bleeding [*n* = 1]), without need for invasive strategy. With regard to device complications, there was no significant difference in the incidence of DRT between the two groups (3.13 vs. 2.50%, *p* > 0.9).

**Table 3:** Clinical outcomes after implantation.

	**Matched cohort**		***P*-value**
	**Thrombocytopenic group (*n* = 32)**	**Control group (*n* = 160)**	
Primary outcomes	4 (12.50)	12 (7.50)	0.313
All cause-death	0	0	≥0.999
Ischemic stroke	0	5 (3.13)	0.592
Systemic embolization	0	1 (0.63)	≥0.999
Major bleeding	4 (12.50)	6 (3.75)	0.065
**Secondary outcomes**			
Minor bleeding	5 (15.63)	2 (1.25)	0.002
Device-related thrombus	1 (3.13)	4 (2.50)	≥0.999

**Figure 2:**
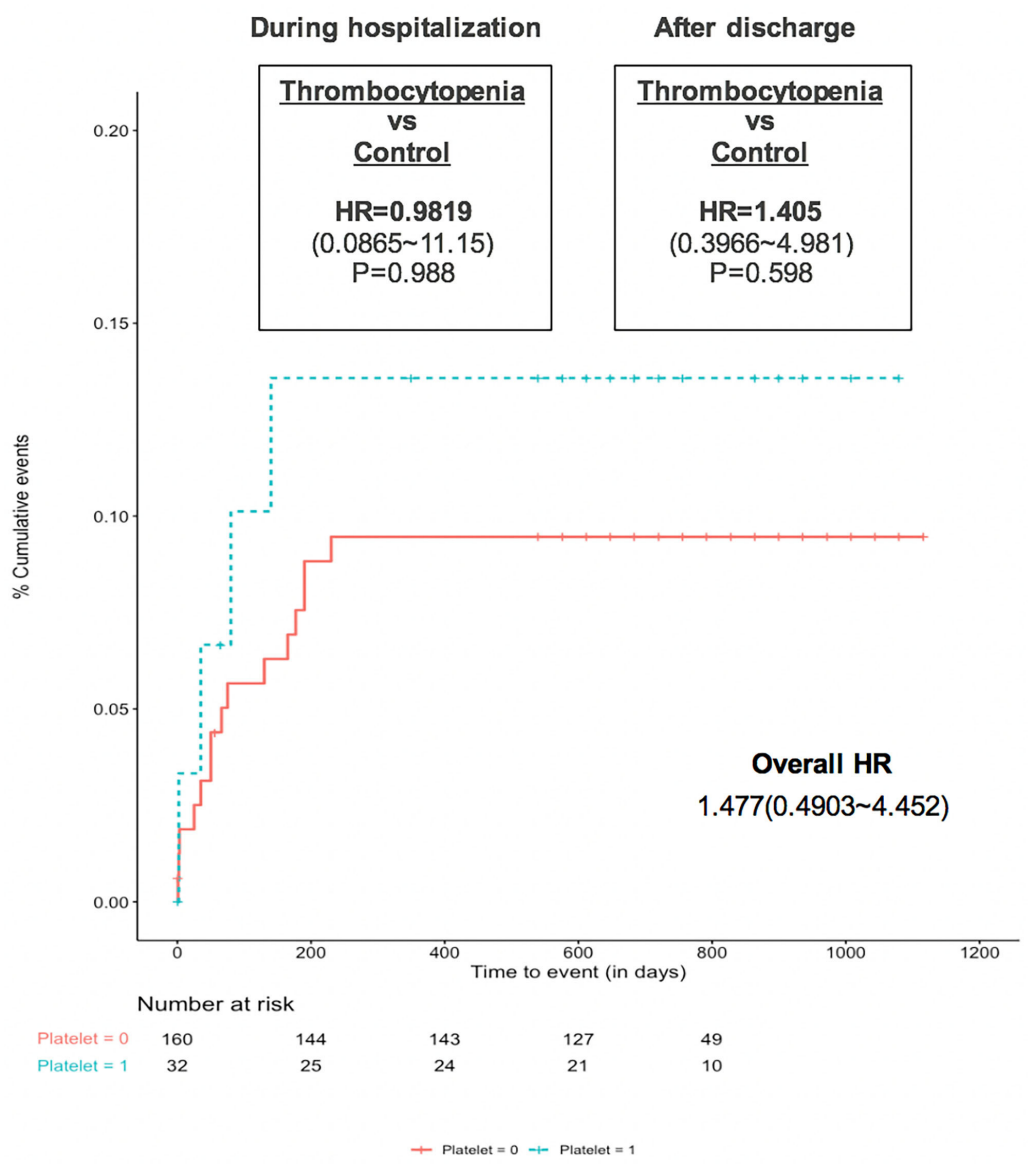
Cumulative incidence of the net adverse event between patients with and without chronic thrombocytopenia (matched cohort).

**Figure 3:**
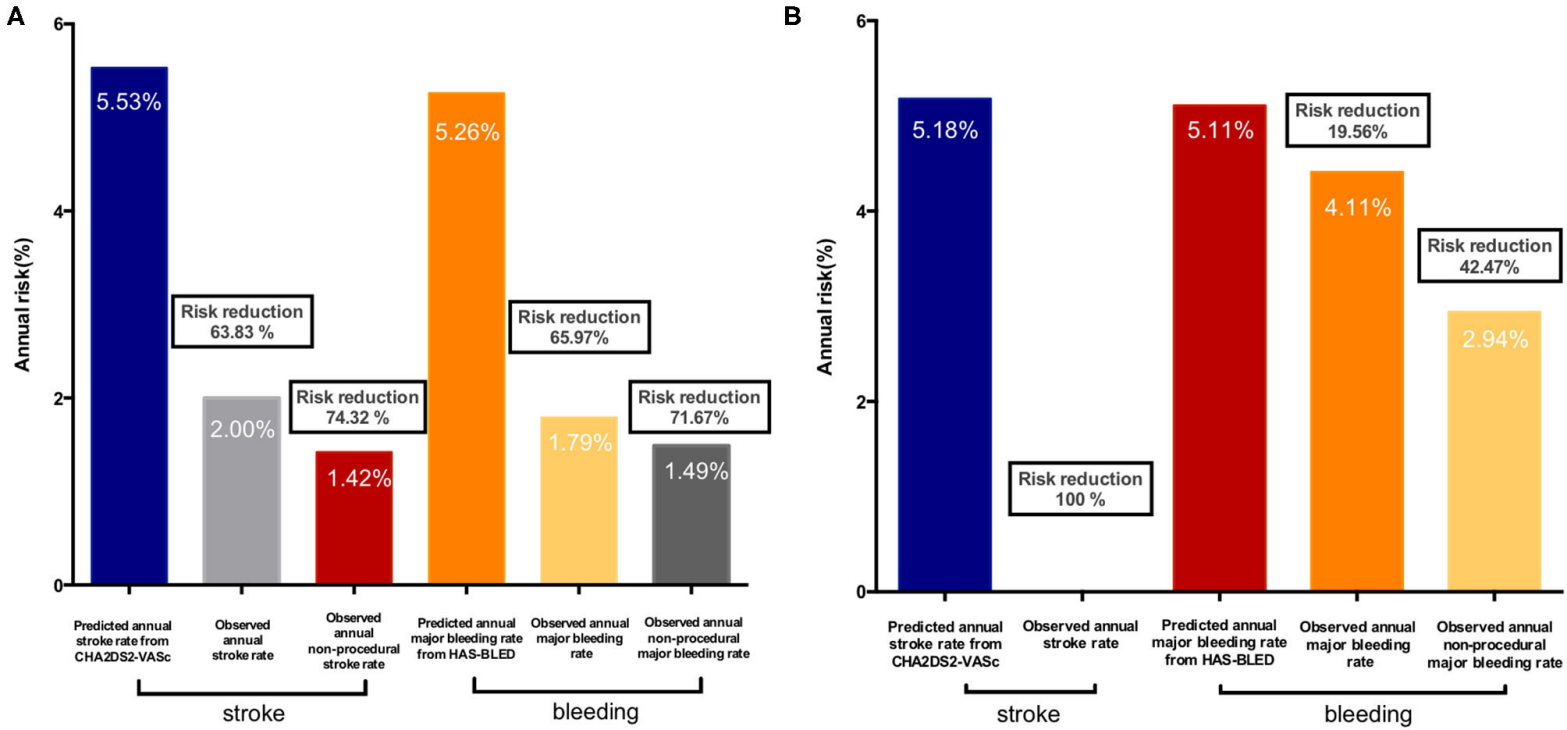
Observed annual rate of thromboembolic events and bleeding events vs. the expected rate based on CHA2DS2-VASC2 score and HAS-BLED risk: **(A)** Control group; **(B)** Thrombocytopenia group.

In terms of the net adverse outcomes, including all major clinical adverse events and imaging abnormality as DRT, no significant difference was observed between the Kaplan-Meier survival curves of the two groups (Figure 2). These similar rates, both during hospitalization and after discharge, initially indicated that LAAO would be of comparable benefit for thrombocytopenic patients as it is for the general AF population. Further findings on the considerable effectiveness of LAAO in reducing clinical thromboembolic and bleeding risks, especially during follow-up (according to the predicted rates in both cohorts) were shown in Figures 3A,B. The observed annual rate of non-procedural stroke and systematic thromboembolism, compared with the value that was predicted using the CHA2DS2-VASC score, decreased by 100% in the thrombocytopenia group and 74.32% in the control group. On the other hand, the observed annual major bleeding rate, compared to the expected rate based on the HAS-BLED score, decreased by 42.47 and 71.67% in the thrombocytopenic and control group, respectively.

### Predictors of Different Endpoints in Our Study Population

cTCP was identified as a strong independent predictor of overall bleeding (OR: 8.150; 95% CI: 2.579~25.757, *p* <0.001) as well as major bleeding complications (OR: 4.571; 95% CI: 1.133~18.438, *p* = 0.033) after LAAO (Table 4). Another important contributor to overall (OR: 2.307, 95% CI: 1.254~4.243, *p* = 0.007) and major hemorrhagic events (OR: 2.441, 95% CI: 1.177~5.061, *p* = 0.016) was patients' HAS-BLED score. Higher absolute platelet count was identified as the only independent predictor of more frequent stroke events in our study cohort (OR: 1.015, 95% CI: 1.002~1.029, *P* = 0.022). Different platelet parameters were also associated with a higher incidence of DRT formation, but this was not indicated to be significant in multivariate analyses. A history of strokes was demonstrated as an independent risk factor for the primary endpoint (OR: 3.00, 95% CI: 1.063~8.468, *p* = 0.038) and rates of DRT (OR: 23.509, 95% CI: 1.261~438.351, *p* = 0.023), in which the presence of PDL was another predictor (OR: 17.005, 95% CI: 1.008~286.902, *p* = 0.049).

**Table 4:** Univariate and multivariate analysis of the stroke, bleeding, and other endpoints.

**Clinical outcomes**	**Univariate**			**Multivariate**		
	**OR**	**95% CI**	***P*-value**	**OR**	**95% CI**	***P*-value**
**Primary endpoint**						
Stroke history	3.000	1.063~8.468	0.038	3.000	1.063~8.468	0.038
SEC	3.032	1.011~9.098	0.048			
**Stroke events**						
Platelet count	1.015	1.002~1.029	0.022	1.015	1.002~1.029	0.022
**DRT**						
Platelet count	1.014	1.001~1.027	0.032			
Stroke history	11.583	1.263~106.198	0.030	23.509	1.261~438.351	0.023
PDL	8.467	0.926~77.389	0.058	17.005	1.008~286.902	0.049
LAA flow velocity	0	0~0.073	0.025			
**Overall bleeding events**						
Platelet count	0.990	0.980~1.000	0.041			
Thrombocytopenia	6.330	2.172~18.471	0.001	8.150	2.579~25.757	<0.001
HAS-BLED score	1.957	1.129~3.392	0.017	2.307	1.254~4.243	0.007
Bleeding history	3.311	1.111~9.868	0.032			
**Major bleeding**						
HAS-BLED	2.200	1.107~4.373	0.024	2.441	1.177~5.061	0.016
Thrombocytopenia	3.667	0.972~13.832	0.055	4.571	1.133~18.438	0.033

*SEC, spontaneous echo contrast; DRT, device-related thrombus; PDL, peri-device leak; LAA, left atrial appendage*.

## Discussion

To our knowledge, this is the first study to specifically evaluate the feasibility of LAAO in thrombocytopenic patients during long-term follow-up. The present study is based on a retrospective, propensity-matched comparison of outcomes following LAAO between patients with cTCP and those with a normal platelet count. The primary findings of this study are: (1) The procedure of LAA implantation could be safely applied in patients with platelet count <10^*^10^∧^9, with a high achievement ratio and infrequent complications; (2) The thrombocytopenic group has a relatively higher risk of major and minor bleeding after LAAO but no significant difference is found in the incidence rates of thromboembolism, DRT, and net adverse outcomes; (3) Performing LAAO in the thrombocytopenic group has shown considerable effectiveness in reducing clinical thromboembolic and bleeding risks compared to the predicted rates; (4) Thrombocytopenia is identified as a strong independent predictor for overall and major bleeding events while patients with a higher absolute platelet count are associated with higher incidence of strokes and DRT.

The use of anticoagulants, whether to administer warfarin or NOACs, and the optimal dosage are controversial in cTCP patients due to the risk of exposing them to long-term bleeding concerns. LAA closure has emerged as a reliable alternative to OAC for the prevention of thromboembolism in AF patients, especially those at high risk for stroke and bleeding with a contraindication to anticoagulants (14). Whereas, AF patients with thrombocytopenia are still totally excluded from current LAAO registry studies owing to the scarcity of data on procedure-related complications and follow-up outcomes in this special population. A previous report by Shokr et al. (15) demonstrated higher risks of vascular injury, bleeding and in-hospital mortality among cTCP individuals in a sample of in-patients undergoing transcatheter valvular intervention and cardiac device implantation. According to these results, the antithrombotic strategy for AF patients with cTCP was caught in a dilemma.

Our study is the first to provide the peri-procedural and long-term outcomes through PS matching patients with and without thrombocytopenia. Substantially, the composite endpoints including clinical thromboembolic and major bleeding events were similar between the two groups. Mortality was not reported in our study and the LAA device was successfully implanted in all patients, suggesting the feasibility of percutaneous LAA closure in cTCP cases, when conducted under close surveillance. However, a detailed analysis of the individual components showed that major bleeding actually occurred more frequently in cTCP patients, as did minor bleeding. Moreover, the annual observed major bleeding rate in our study's cTCP group was higher than the 1.5–3.5% (16, 17) stated in other registry investigations, even though non-procedural bleeding was assessed separately. Stissing et al. (18) reported that platelet function, as tested by multielectrode aggregometry (MEA), was impaired when platelet count was lower than 150^*^10^∧^9 000 /L. Hanke et al. (19) also found that a platelet count of <100^*^10^∧^9/L was associated with a decrease in platelet function of 18%, while Ranucci et al. (20) posits a stronger role for platelet count, suggesting that it could directly predict post-operative bleeding independent of platelet function. Our further analysis indicating an inverse relationship between quantitative platelet activity and bleeding events was in agreement with these findings (21, 22), deriving from other surgical or percutaneous treatment, that bleeding was still a concerning complication in cTCP patients. Notably, 4/4 major bleeding events and 4/5 minor bleeding events in the cTCP group occurred during anticoagulation or DAPT therapy, while only one gingival bleeding occurred during ASA therapy at 1-year follow-up. Aside from cTCP patients experiencing a relatively lower reduction of bleeding risk than the control group, it was important that LAAO still demonstrate a significant decrease in the observed rate of non-procedural bleeding compared to the predicted values. Patients undergoing successful LAA closure without serious residual leak are typically treated with ASA monotherapy after ~6 months of intensive pharmacotherapy (23). Therefore, we could more readily postulate as to the long-term benefits of LAAO if our study period were extended to when patients are able to discontinue OACs and DAPT.

In the current study, cTCP patients had comparable rates of clinical thromboembolism to the control group, but the occurrence of stroke was independently less frequent in patients with a lower absolute platelet count. Our result was consistent with the conclusion in other studies (24, 25) that patients who presented with ischemic strokes showed significantly higher platelet counts at baseline. It is also well-acknowledged that AF-related hemodynamic abnormality can result in blood stasis in the left atrium, elevated intra-atrial pressure, and histological lesions in the endothelial lining of the atrial walls, followed by aggregated platelets releasing inflammatory mediators and substances to facilitate coagulation (26, 27). Hence, when the total count of circulating platelets is higher, a greater number of platelets can be engaged at the site of initial reaction, leading to faster thrombosis (28). In spite of the remarkable reduction in thromboembolic events compared to the predicted rates in our study, we also suggest that the clinical profile of our study should be interpreted dialectically, taking into consideration the real-world risks for cTCP patients with AF with regard to being under medication or without prophylaxis.

Device-related thrombus is another dominant concern for LAA implantation. In our patients we saw a relationship between higher platelet count and the prevalence of thrombus formation on the device, similar to the relationship observed in the study by Plicht et al. (29) based on patients undergoing LAAO using the Amplatzer Cardiac Plug. As for DRT, the platelet-induced thrombus growth taking place during the vulnerable device endothelialization period is what emphasizes the significance of antiplatelet therapy (30). An interesting finding in our cohort with regard to DRT was that they were all undergoing antiplatelet therapy at the time of DRT detection, which indicated that the protection provided by DAPT inhibition was insufficient. It should be noted that the only cTCP patient to develop DRT had been prescribed with DAPT therapy directly post-procedure due to his intolerance of NOACs before admission. However, he still experienced an upper gastrointestinal bleeding event at 35 days after discharge. He was then treated with ASA monotherapy, upon which an unexpected DRT occurred. Therefore, this conflicting case enlightened us to re-examine the optimal evaluation of eligible cTCP patients for LAAO. Since none of the main major adverse events in our study occurred beyond 1 year after implantation, in the future, a larger study with a longer follow-up period and a high degree of procedural safety will be vital to determine the overall risk-benefit ratio of LAAO in AF patients with cTCP.

## Limitation

This is a single-center, non-randomized study based on a small sample size. Despite PS matching, the retrospective and non-randomized nature of the study could not totally correct the erroneous omissions or unmeasured confounders in baseline characteristics and clinical outcomes of our study population. Meanwhile, the propensity matching ratio of our study was defined as 1:5, hence, the limited patients in the cTCP group might also result in certain selection bias and influence the statistical power. All the data of our study population was obtained and analyzed retrospectively and therefore, to some degree, our research lacked standardization in comparison to other registry studies. Furthermore, the limited volume of LAAO procedures only using Watchman devices also indicated that our conclusions might not be extrapolated to other LAA devices.

## Conclusion

Among a well-matched LAAO population, successful implantation was achieved in all cTCP patients, who developed the composite endpoint of thromboembolism and bleeding events at a comparable rate to those without cTCP during a mid- to long-term follow-up. However, lower platelet count was found to be associated with a higher incidence of bleeding complications and a decreased risk of stroke or DRT. Consequently, larger randomized studies are needed to continue exploring the definite impacts of LAAO in AF patients with thrombocytopenia.

## Data Availability Statement

The raw data supporting the conclusions of this article will be made available by the authors, without undue reservation.

## Ethics Statement

The studies involving human participants were reviewed and approved by the Institutional Review Board of Zhongshan Hospital, Fudan University, Shanghai, China. The patients/participants provided their written informed consent to participate in this study.

## Author Contributions

XZ, QJ, and JH worked on the conception and organization of the research project. Material preparation, data collection, and analysis were performed by QJ, DC, SC, and ML. QJ wrote the first draft of the manuscript. XZ, DK, CP, DZ, and JG reviewed the manuscript. All authors read and approved the final version of the manuscript.

## Conflict of Interest

The authors declare that the research was conducted in the absence of any commercial or financial relationships that could be construed as a potential conflict of interest.
